# 825. Cryptococcosis in the Immunocompetent Patient: Incidence and Outcomes

**DOI:** 10.1093/ofid/ofad500.870

**Published:** 2023-11-27

**Authors:** Valli Mani, Adam Zimilover, Ji-Cheng Hsieh, Maham Ghani, Simon Kashfi, Richie Chen, Iffath Islam, Henry Donaghy

**Affiliations:** Donald and Barbara Zucker School of Medicine at Hofstra/Northwell, Forest Hills, New York; Donald and Barbara Zucker School of Medicine at Hofstra/Northwell, Forest Hills, New York; Donald and Barbara Zucker School of Medicine at Hofstra/Northwell, Forest Hills, New York; Donald and Barbara Zucker School of Medicine at Hofstra/Northwell, Forest Hills, New York; Donald and Barbara Zucker School of Medicine at Hofstra/Northwell, Forest Hills, New York; Donald and Barbara Zucker School of Medicine at Hofstra/Northwell, Forest Hills, New York; Donald and Barbara Zucker School of Medicine at Hofstra/Northwell, Forest Hills, New York; Northwell Health, New Hyde Park, New York

## Abstract

**Background:**

Cryptococcosis is a fungal infection that can cause pulmonary, central nervous system, or disseminated disease in immunosuppressed individuals. Although pulmonary cryptococcosis is known to rarely affect immunocompetent patients, there is limited research comparing the incidence and clinical outcomes of cryptococcosis in immunocompetent versus immunocompromised patients.

**Methods:**

A retrospective analysis was performed on patients with confirmed positive CSF PCR, CSF antigen, or serum cryptococcal antigen tests from 8 hospitals in New York from 2017-2022. Patients were classified as immunocompromised if they had HIV, an organ transplant, active cancer, or were on immunosuppressive medications at the time of diagnosis. Baseline demographic information, comorbidities, and clinical outcomes were recorded. Statistical methods performed include t-test, Chi-square, and Fisher exact test.

**Results:**

37 cases were identified with 9 cases (24%) in immunocompetent patients. There were no significant differences in age (p=0.82), gender (p=0.61), or BMI (p=0.15) amongst immunocompetent vs immunocompromised patients. Immunocompetent patients were more likely to have diabetes compared to immunocompromised patients (44% vs 21%, p = 0.04). Immunocompetent patients presented with meningitis more frequently (78% vs 41%, p = 0.05) with decreased mortality (11% vs 18%, p = 0.04) and no significant differences in rates of fungemia (21% vs 22%, p = 0.28). The majority of immunocompetent patients were treated inpatient and discharged on fluconazole without documented relapsed infection (77%). When assessing patients before and after 3/30/2020, the midpoint of our data timeline and beginning of the COVID pandemic, the proportion of immunocompetent cases increased (12% vs 35%, p = 0.03) while the total number of cases was relatively unchanged.

**Conclusion:**

Meningitis was a more common presentation than pulmonary disease in immunocompetent patients. Immunocompetent patients also had better outcomes with decreased mortality. Overall, the number of immunocompetent patients increased over the study period. Additional information about cryptococcosis in immunocompetent patients, as well as the role of diabetes and COVID infection as potential risk factors, warrants further study.

**Disclosures:**

**All Authors**: No reported disclosures

